# Differences in timing of mating swarms in sympatric populations of *Anopheles coluzzii* and *Anopheles gambiae s.s.* (formerly *An. gambiae* M and S molecular forms) in Burkina Faso, West Africa

**DOI:** 10.1186/1756-3305-6-275

**Published:** 2013-09-22

**Authors:** Simon P Sawadogo, Carlo Costantini, Cédric Pennetier, Abdoulaye Diabaté, Gabriella Gibson, Roch K Dabiré

**Affiliations:** 1 Institut de Recherche en Sciences de la Santé (IRSS)/Centre Muraz, Bobo-Dioulasso 01 BP 545, Burkina Faso; 2 IRD/UMR, MIGEVEC (UM1, UM2, CNRS5290, IRD 224), Centre IRD de Montpellier, Montpellier Cedex 5 BP 64501, 34394, France; 3 IRD/CREC, MIGEVEC (UM1, UM2, CNRS5290, IRD 224), Cotonou 08 BP 841, Bénin; 4 Department of Agriculture, Health and Environment, Natural Resources Institute (NRI), University of Greenwich at Medway, Chatham ME4 4TB, Kent, UK

**Keywords:** Activity rhythms, Actographs, Allochronic speciation, Behaviour, Circadian rhythms, Environmental factors, Mating swarms, Reproductive isolation, *Anopheles coluzzii*, *Anopheles gambiae s.s*

## Abstract

**Background:**

The M and S molecular forms of *Anopheles gambiae s.s.* Giles appear to have speciated in West Africa and the M form is now formally named *An. coluzzii* Coetzee & Wilkerson *sp.n.* and the S form retains the nominotypical name (abbreviated here to *An. gambiae*). Reproductive isolation is thought to be the main barrier to hybridisation; even though both species are found in the same mating swarms, hybrid fertilisations in *copulae* have not been found in the study area. The aim of the study, therefore, was to determine whether differences in circadian and/or environmental control over the timing of swarming in the two species contribute to reproductive isolation.

**Methods:**

The timing of male swarming in these species was recorded four nights per month over four years at five swarming sites in each of two villages. The timing of the start and end of swarming, and the concurrent environmental parameters, temperature, humidity and light intensity, were recorded for n = 20 swarms/month/species. The timing of 'spontaneous’ activity at dusk of individual *An. coluzzii* and *An. gambiae* males was video-recorded in an actograph outdoors for 21 nights.

**Results:**

Of the environmental parameters considered, swarming was most strongly correlated with sunset (r^2^ > 0.946). *Anopheles gambiae* started and stopped swarming earlier than *An. coluzzii* (3:35 ± 0:68 min:sec and 4:51 ± 1:21, respectively), and the mean duration of swarming was 23:37 ± 0:33 for *An. gambiae* and 21:39 ± 0:33 for *An. coluzzii.* Accordingly, in principle, whenever both species swarm over the same marker, a mean of 15.3 ± 3.1% of *An. gambiae* swarming would occur before *An. coluzzii* males arrived, and 19.5 ± 4.55% of *An. coluzzii* swarming would occurred after *An. gambiae* males had stopped swarming. These results are consistent with the finding that *An. gambiae* males became active in the actograph 09:35 ± 00:22 min:sec earlier than *An. coluzzii* males*.*

**Conclusions:**

The timing of swarming and spontaneous activity at dusk are primarily under circadian control, with the phase linked closely to sunset throughout the year. The mating activity of these two species is temporally segregated for 15-20% of the swarming period, which may contribute to the observed reproductive isolation of these species in local sympatric populations.

## Background

Despite concerted international efforts to reduce malaria worldwide, in sub-Saharan Africa alone there were > 600,000 deaths in 2010 attributed to this deadly disease, and more than half the world’s population is still at risk of malaria infection [1]. In the absence of an effective vaccine, long-lasting insecticide-treated nets (LLINs) and indoor residual spraying (IRS) are the most common methods used to control malaria [2]. However, the success of malaria vector control programmes is threatened by the emergence and spread of insecticide resistance to all major classes of insecticides, including pyrethroids, and to a lesser degree carbamates and organophosphates [3-7]. Alternative novel vector control strategies, such as those based on genetically modified mosquitoes (GMO) or the release of sterile males [8-12], are being investigated to explore their usefulness for sustainable long-term malaria control. The application of current control strategies and the prospects of using innovative control tools, however, crucially depend on accurate knowledge of the bionomics of the vectors, with particular reference to their reproductive biology, population structure and circulation of genes of interest in natural field populations.

The major afro-tropical malaria vector *Anopheles gambiae s.s.* Giles represents a challenging model in this context, because its composite population structure is complicated by the emergence of independent reproductive units marking a process of incipient speciation. Two such units, originally referred to as 'M’ and 'S’ molecular forms and now formally recognised as *An. coluzzii* Coetzee & Wilkerson and *An. gambiae s.s*. Giles (hereafter *An. gambiae*) based on population genomic evidence [13], appear to be following independent evolutionary trajectories [14], diverging at the genetic and ecological levels despite ongoing, albeit limited, gene flow [13]. Across most of West Africa, the frequencies of hybrids of the two species are < 1% [15]. Current hypotheses point to a process of ecological speciation, whereby a combination of divergent ecological and behavioural characteristics have contributed to reduced gene flow and nearly complete reproductive isolation [13].

The exact nature of the reproductive barrier between *An. coluzzii* and *An. gambiae*, which is substantiated, with a few exceptions [16-19], by the rarity of hybrids in natural field populations [15], is not fully established. There is evidence that pre-mating isolation must play a significant role; in a zone of sympatry in Mali, only 3 out of 250 (1.2%) wild caught *An. coluzzii* and *An. gambiae* females were found to contain the sperm of the 'other’ species [20], and in Burkina Faso no hybrid fertilisations have been found in *copulae* caught in mixed-species swarms (i.e., swarms consisting of both *An. coluzzii* and *An. gambiae* males). Although the occasional mixed-species *copulae* have been caught, in all cases the females contained sperm only of the female’s species [21].

It is generally agreed that swarming in mosquitoes at species-specific sites and times of day facilitates mating [22-27] and that males and virgin females are attracted to swarm sites by visual features in the landscape. Upon reaching a swarm site, each mosquito maintains a looping flight pattern, station-keeping in relation to a visual 'marker’, until a potential mate is detected, whereupon a mating chase ensues [22,28,29]. Mosquito swarms differ from other types of swarms (e.g., of bees or termites) in that swarm cohesion depends primarily on individuals responding in the same way to stationary markers, and to a lesser extent on interactions between individuals [30]. Hence, a single mosquito can be said to 'swarm’, since it will continue to hold station with respect to the marker in the absence of other mosquitoes. Although the daily timing of swarm formation and site choice tends to be associated with particular species, different species may swarm in the same area, although they generally form separate swarms at varying distances from the marker [27].

*Anopheles gambiae* has been shown in the laboratory to mate within the first hour of darkness, during a peak in flight activity, and when males are most responsive to the flight tones of females [31]. Field studies of *An. gambiae* swarming have focused primarily on flight patterns and on the close association between swarming and mating [32-35].

Recent studies on swarming and mating behaviour in *An. gambiae s.l.* in Mali and Burkina Faso have targeted differences in the behaviour of *An. coluzzii* and *An. gambiae* that appear to have contributed to a premating barrier to hybridisation in some locations [21,34,36-39]. Studies in Mali have shown that the lack of hybridisation between *An. coluzzii* and *An. gambiae* could be explained, in part at least, by form-specific differences in choice of swarming sites, i.e., spatial segregation of mating sites [38,39]. In a previous study conducted at the same sites in Burkina Faso as the study described here, out of 90 swarms monitored over 2 years, 60% were single-species swarms of necessity, as the species were segregated either spatially or seasonally (i.e., did not occur in the same villages during the same months), 23% were single species swarms even though both species were present in the village and only 17% were 'mixed-species’ swarms. Of the 33 females caught in *copulae* collected from these mixed-species swarms, all were found to be inseminated by males of their own species [21], suggesting that there are as yet unidentified mechanisms preventing hybrid matings within mixed-species swarms. One of these mechanisms could be slight differences in the start and/or end times of swarming on a given day, i.e., daily, as opposed to seasonal, temporal mating segregation. In the previous study [21], swarms were sampled during the daily peak time of swarming, a sampling regime that would not have detected differences between species in the timing of their swarming.

The studies referred to above [21,38,39] focused on factors that affect *where* swarming takes place. The factors that could affect *when* swarming occurs, however, remain poorly investigated, and, therefore, are the main focus of the study presented here.

Generally, the timing of mosquito behaviour is mediated by circadian rhythms of activity that are controlled by endogenous factors, and to a lesser degree in response to environmental cues, such as changes in temperature, humidity and light intensity [27,40]. Male and virgin female *An. gambiae* have been shown to express similar patterns of daily 'spontaneous’ activity (i.e., flight in the absence of environmental stimuli), with a major peak during the first hour after 'lights off’ in the laboratory (i.e., at sunset) and a smaller peak at 'lights on’ (sunrise), that coincide with the observed timing of swarms in the field [27,41].

Little is known, however, about the relative timing of swarming behaviour in *An. coluzzii* and *An. gambiae* in the field. Therefore, to test the hypothesis that *An. coluzzii* males start and/or end swarming at different times to *An. gambiae* males, we recorded the timing of swarming activity of each species at natural swarm sites and the timing of spontaneous activity levels of individual *An. coluzzii* and *An. gambiae* males at dusk in an actograph; in both cases the mosquitoes were under the influence of both their endogenous circadian rhythms of spontaneous activity and natural changes in potential environmental cues. We recorded the timing of the start and end of swarming activity at dusk, the concurrent environmental parameters that might affect the timing of swarming in the field (temperature, humidity, light intensity and the time of sunset) to assess the relative importance of endogenous and environmental factors in the control of the timing of swarming in *An. coluzzii* and *An. gambiae.*

We also recorded the timing of spontaneous activity at dusk, which would normally initiate the egress of mosquitoes from their day-time resting sites [27], thereby exposing them to environmental cues related to a range of behaviours, e.g., visual and possibly auditory and olfactory cues that guide them to swarming sites and/or sources of nectar. Small differences in the timing of activation could have a profound effect on the chances of males and virgin females of the two species encountering each other during the short window of time each day when mating occurs.

## Methods

### Study sites

**Vallée de Kou** (11°24’29”N; 04°24’37”W) consists of a cluster of villages ~30 km north-west of Bobo-Dioulasso, in the valley of the Kou River, a region of extensive rice cultivation that was established during the 1970s. Seven villages covering 7,200 ha were created as part of an irrigation development scheme. Each village lies at the edge of rice fields and is surrounded otherwise by wooded savannah. Since the Kou River flows all year, it offers a permanent source of water for irrigation, enabling the growth of two crops of rice per year (July-November and January-May), and as a result the rice fields are highly productive permanent mosquito breeding sites. More typical anopheline breeding sites (rain puddles and rain or ground-water filled depressions, such as tyre tracks) are also present. Both *An. coluzzii* and *An. gambiae* have been recorded at high densities during the rainy season (May-October), with typical biting rates for *An. coluzzii* of ~ 200 bites person^–1^ night^–1^[42]. Since 2003 various attributes of *An. coluzzii* and *An. gambiae* mating swarms have been monitored in the village known as 'VK7’ and a range of associated environmental and ecological parameters have been recorded, as described by [36].

**Soumousso** (11°00’46”N, 4°02’45”W) is a typical Guinean savannah village situated ~ 55 km east of Bobo-Dioulasso. The main anopheline breeding sites are rain puddles and a semi-permanent swamp. Three main malaria vectors are found in this village *An. coluzzii, An. gambiae*, *An. funestus* Giles and *An. nili* (Theobold). *Anopheles arabiensis* Patton, another member of the *An. gambiae s.l.* species complex, is occasionally reported at low frequencies (<5% of *An. gambiae s.l.* samples). Since 2003 the swarming behaviour of *An. coluzzii* and *An. gambiae* have been studied in this village [21,37].

### Monitoring the timing of *An. coluzzii* and *An. gambiae* swarming

Since 2005 male swarms in the two study villages have been monitored with the aim of characterising swarming sites and monitoring the identity of males collected from each swarm. The main findings of the earliest surveys (2005- early 2006) were that *An. coluzzii* swarms predominate in Vallée de Kou, whereas *An. gambiae* swarms predominate in Soumousso, and specific swarm sites were identified where only *An. coluzzii* or *An. gambiae* swarms occurred [21]. On the basis of these data, for the present study five swarm sites were chosen in each village that were most likely to have single-form swarms of *An. coluzzii* in Vallée de Kou and *An. gambiae* in Soumousso.

Trained IRSS/Centre Muraz field teams (> 5 years experience) monitored the five selected swarm sites in each village on four consecutive days each month for a year from January to December 2007, and each month from August to October 2006, and June-Oct in 2008 and 2009, with no exceptions due to weather or any other factors. Swarm observations were made from 17:00 to 20:00. The number of swarms monitored each month was: two species × five swarms/species × four observations of each swarm/month = 40 swarms observed/month × 25 months, i.e., a total of 1,000 swarms.

The start of swarming was defined as the time the first male was seen flying in characteristic swarming flight at the specified swarm sites. It was observed that, after the first male arrived, there was a rapid accumulation of males in swarms, which eventually consisted of tens to thousands of males, depending mainly on seasonal variation in population densities [21]. The total number of males was not static; individual males appeared to leave and join the swarm throughout the swarming period. Toward the end of this period, the number of males appeared to decrease as quickly as they had accumulated. By the end of the swarming period it was too dark to see every mosquito clearly with the naked eye. Therefore, to be sure the end of swarming was recorded accurately, as determined by the departure of the last mosquito, a camera flashbulb was lit ~ once per minute near the expected end of swarming; single mosquitoes are clearly visible in the brief but high intensity light of the flash, which is too brief to disturb swarming behaviour. The only exception to the observation regime described in the previous paragraph is that the end of swarming was not recorded in 2009.

Ideally, the time at which each mosquito entered and left the swarm should have been recorded, along with its molecular form identity and the total numbers of males swarming each evening. In practice, however, equipment to continuously record the numbers of males present was not available, and it would be difficult to sample each swarm more than once an evening without disrupting their natural behaviour.

### Mosquito sampling and identification

At least 30 males from each swarm were collected by a single sweep with a sweep net at the estimated peak of the swarming period, as for the surveys in 2005 and early 2006 [21]. Samples were identified to species and molecular form by the PCR method described by Favia *et al.*[21]. The identity of mosquitoes in swarm sites where single-species swarms had occurred in 2005 and early 2006 continued to be monitored for this study August-October of 2006, throughout 2007 and July-October of 2008 and 2009. The identities of at least 30 males per swarm were assessed for all the swarms used in the study presented here from 2006 – 2009, and none were found to consist of more than one species. It must be noted, however, that it is not possible to be absolutely certain a given swarm consists of only a single species without collecting the entire swarm, which would defeat the purpose of recording swarm duration. We have, however, undertaken a standardised sampling protocol and identified a reasonable number of mosquitoes from each swarm; 30 mosquitoes from each of 1,000 swarms (see '**Monitoring the timing of*An. coluzzii*and*An. gambiae*swarming**’).

#### PCR protocol

Genomic DNA (~10-50 ng) from individual *An. gambiae s.l.* specimens was PCR amplified using primers R3, R5, Mopint and B/Sint [43]. The PCR conditions were 30 s at 94°C, 30 s at 63°C and 30 s at 72°C for 25 cycles, with a final extension step at 72°C for 7 min. Amplification products were run in a 1.5% agarose gel. The few specimens (~1.1%) that failed to be identified by this protocol were further analysed by the PCR technique of Scott *et al*. [44], which reliably detects *An. arabiensis,* a sibling species in the *An. gambiae s.l*. complex*.* Only half of these specimens were found to be *An. arabiensis*. The remaining unidentified specimens were reanalysed by the PCR method of Fanello *et al*. [45], and were confirmed to be *An. coluzzii*. We are confident in our choice of methods, in spite of the potential errors raised by Santolamazza *et al.*[46,47], since the risk is an overestimate of hybrid *An. coluzzii*/*An. gambiae* females, but we only identified males.

### Recording environmental parameters in the field

The environmental parameters air temperature (T°), relative humidity (% RH) and light intensity (W/m^2^) were recorded on a data logger (Model: ETHG912, OREGON Scientific, Tualatin, Oregon, U.S.A.) at the start of every swarm observed in 2007. One such data logger was placed on the ground at each swarming site one hour before swarm formation was expected. The sensors were placed on top of the data logger (~ 10 cm above ground), where they would not disrupt the flight of swarming mosquitoes, which occurred from ~ 1 – 2 m above ground. The IRSS/Centre Muraz field teams were responsible for checking that the data loggers ran smoothly and geo-referenced each swarm site to be certain the same sites were used on each occasion.

### Recording individual male activity at dusk under ambient environmental conditions

**Figure 1:**
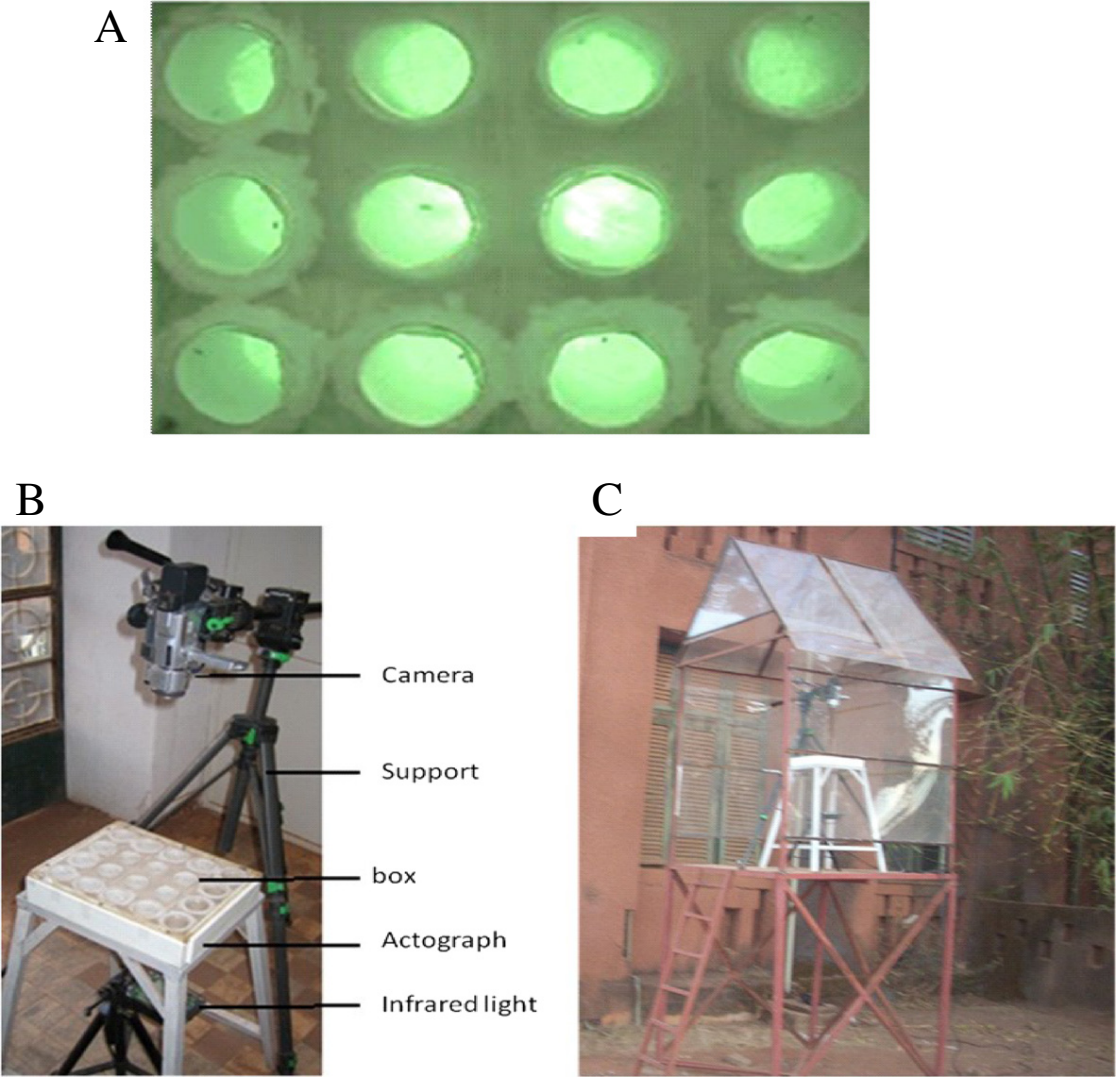
**Actograph equipment to measure onset of spontaneous activity of individual male mosquitoes at dusk.A)** arrangement of chambers that each hold a single mosquito, **B)** arrangement of video camera, actograph frame and infra-red lights, **C)** position of actograph apparatus within a protective framework, but exposed to ambient light, temperature and humidity.

The timing of dusk activity of individual *An. coluzzii* and *An. gambiae* males was recorded outdoors at IRSS/Centre Muraz, Bobo-Dioulasso (Figure 1) to assess the relative importance of endogenous cues (circadian rhythms) and environmental cues (changes in temperature, humidity and light intensity) in controlling the onset of dusk activity. Male mosquito behaviour was recorded continuously from 17:00 to 20:00 on 21 evenings in September and October, 2008 with an actograph that measures the timing of general activity of individual insects isolated from each other [48]. The actograph consists of 12 chambers (6.5 cm deep, 3.5 cm diameter), arranged in rows of 3 × 4 in a Perspex frame (Figure 1A). A single male was placed in each of the 12 chambers (six *An. coluzzii* and six *An. gambiae* males each evening). Each mosquito was isolated from visual, auditory and olfactory cues from the other mosquitoes that might stimulate activity (a layer of sand surrounded each of the 12 chambers; note dark areas between chambers in Figure 1A). The bottom of each chamber was covered with clear Perspex and the tops were covered by a thin sheet of clear plastic, so that mosquitoes were exposed to ambient light intensity. A set of meterological sensors was placed next to the lid of the actograph box (Figure 1B) to record the air temperature, humidity and light intensity at the level of the actograph. The protective layer of clear plastic on top of the apparatus was not thought to have a significant 'greenhouse’ effect on the temperature in the actograph chambers since the experiment began within 1 hour of sunset, by which time the apparatus was fully shaded by the nearby building. The shelter (Figure 1C) was open to air flow and the actograph was not sealed.

The male mosquitoes were the offspring of gravid females collected from the wild in Vallée du Kou (*An. coluzzii*) and Soumousso (*An. gambiae*) and reared under the same environmental conditions in the insectary at IRSS/Centre Muraz. To ensure that the males were sexually mature and a standardized age, only 3-day-old males were used.

Six males of each species were randomly selected, placed in the actograph ~ 1 hour before sunset and their behaviour was video-recorded for a single 3 hour dusk period (17:00 – 20:00) at IRSS/Centre Muraz (Figure 1). The placement of *An. coluzzii* and *An. gambiae* males in the actograph was varied daily to control for position.

The behaviour of each of the 12 mosquitoes in the actograph was recorded with a video camera (SONY Model: DCR-TRV60E; Figure 1B) installed on a platform 2 m above ground, where the actograph was exposed to ambient environmental conditions (Figure 1C). The video camera was positioned above the actograph looking down, so that the mosquitoes in all 12 chambers were visible (Figure 1C). A bank of infra-red lights (900 nm wavelength LEDs; at the peak sensitivity of the video camera, but beyond the mosquito’s spectral sensitivity [49] was positioned under the actograph (Figure 1B) to provide even lighting for the video camera throughout the steep change in ambient light intensity across dusk. The intensity of infra-red light was high enough to compensate for changes in ambient illumination throughout dusk, so that the recorded video image remained at a constant brightness throughout the recording period.

The timing of spontaneous activity was recorded for n = 126 males of each species (6 males of each form/night, for 21 nights in September – October, 2008). The data on timing of activity of each group of mosquitoes in the actograph was determined by Security Spy (http://www.bensoftware.com/securityspy|) software installed on a Mac G5 computer.

### Data analysis

Pearson’s Correlation Coefficient Test, based on critical values for the regression 'r’ (two-tailed distributions), was used to assess the correlation between each environmental variate in turn (temperature, humidity, light intensity and time of sunset) with the start and end of swarming times, with the statistical package 'R’ [50]. The distribution of all variates was found to be normal by visual observation of histograms. The squared correlation coefficient (r^2^) is also reported as a measure of how much of the variability in swarm times can be explained by a particular variate, e.g., if r^2^ = 0.54, it is likely that 54% of the correlation can be accounted for by that variate.

Unfortunately, it is not possible to compare the mean swarm start and stop times of the two species directly because the data for *An. coluzzii* were collected only in VK7 and for *An. gambiae* only in Soumousso since site and species are confounded. The cause of statistically significant differences could be due to differences between the villages and/or between species. Indeed, since the villages are situated ~ 24' latitude and ~ 22’ longitude apart, the clock times at sunset differed by an average of 1:30 (min:sec) throughout the year, varying within a range of 0:46 (December) to 2:12 (June) due to the tilt of the Earth and its irregular shape. Accordingly, even if the two species started swarming at the same 'time of day’ in relation to sunrise/sunset, on average *An. gambiae* in Soumousso would begin to swarm ~1:30 min:sec before *An. coluzzii* in VK7 by clock time. Therefore, for comparisons between the two species, swarming start and end times were indexed to local sunset times at their respective sites by calculating the offset between the local start time or end time of swarming and the local time of sunset on the same evening. The mean monthly differences between the two species in relation to the time after sunset swarming started or ended, and swarm durations were compared by Welch’s *t*-test (unequal variances).

To analyse the actograph recordings, the 3 hour dusk observation period was divided into 180–1 minute 'time bins’, and each mosquito was given a score of '1’ if it was active for at least 25% of the minute, or '0’ otherwise. The proportion of mosquitoes active out of the total possible (126 male for each species) was calculated for each species for each minute. The time at which 50% of males were active was calculated with a probit-like 'LD50’ GLM analysis, modified by fitting a 'cauchit’ transformation (similar to a probit, but with more weight on the tails of the distribution) [50]. An ANOVA was used to test sequentially the effect of time, form and time:form on the distribution of data points.

## Results

### Species composition of swarms

We genotyped 30 mosquitoes per swarm to determine their species and molecular form identity. As expected, the samples from Vallée du Kou were composed exclusively of *An. coluzzii* and those from Soumousso were exclusively *An. gambiae*.

### Effects of environmental parameters on swarm times

**Figure 2:**
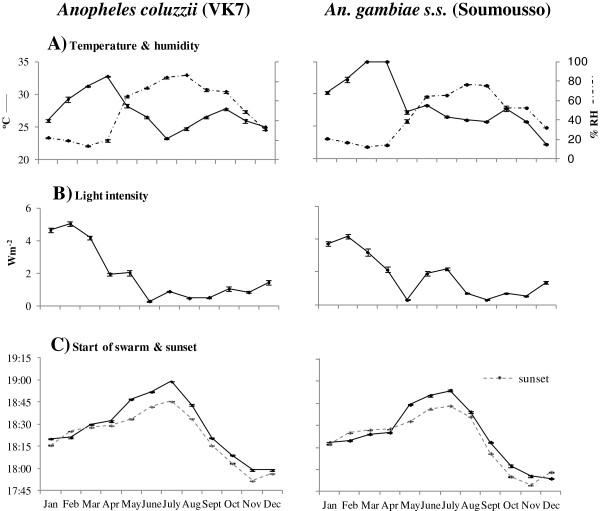
**Relationships between the start of male swarming for *Anopheles coluzzii *(in VK7) and *An. gambiae s.s. *(in Soumousso) and environmental conditions. A)** Mean ± standard error (s.e.) temperature (solid line, left axis) and humidity (dashed line, right axis) when first male(s) observed swarming, **B)** light intensity at start of swarming and **C)** time at which first male(s) observed swarming (solid line) and time of sunset on swarm monitoring days. For each data point n = 20 (for each species, the same five swarm sites monitored on four consecutive days each month). Note: tick marks on the x-axis mark the boundaries between months.

**Figure 3:**
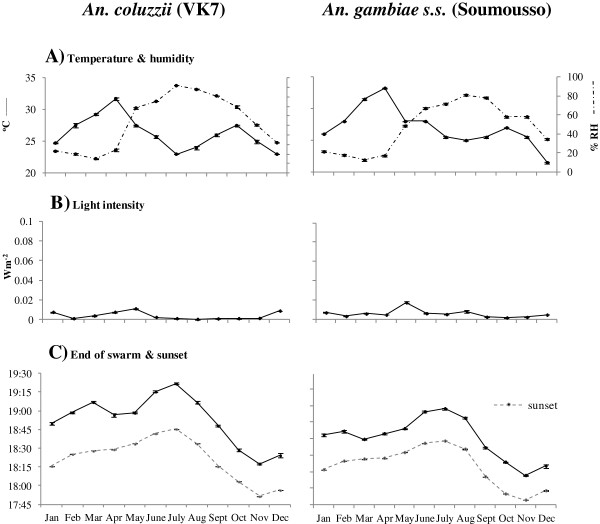
**Relationships between the end of male swarming for *Anopheles coluzzii *(in VK7) and *An. gambiae s.s. *(in Soumousso) and environmental conditions. A)** Mean ± standard error (s.e.) temperature (solid line, left axis) and humidity (dashed line, right axis) when last male(s) observed swarming, **B)** light intensity at end of swarming and **C)** time at which first male(s) observed swarming (solid line) and time of sunset on swarm monitoring days. For each data point n = 20 (for each species, the same five swarm sites monitored on four consecutive days each month).

The mean monthly temperatures and humidities at the start and end of swarming are shown in Figures 2A and 3A, respectively. The overall patterns across the months reflect seasonal changes; with higher temperatures and lower humidities in the dry season (February-April), cooler temperatures and higher humidities in the rainy season (June-October) and sharp transitions in May and October. Monthly mean light intensities at the start and end of swarming are shown in Figures 2B and 3B, respectively. Light intensities at the start of swarming generally decreased from January to December, but varied little at the end of swarming. The mean monthly times of the start and end of swarming are shown in Figures 2C and 3C, respectively, with the associated mean monthly times of sunset on the days swarm times were recorded.

A regression analysis of swarm start times on the concurrent temperature, humidity or light intensity, in turn, found that humidity had the strongest correlation with start times for both species, explaining 19.8% of variation in swarm start times for *An. coluzzii* and 8.0% for *An. gambiae* (Pearson’s Correlation Coefficient Test, p < 0.001, Table 1). Humidity was also strongly correlated with the end of swarming for *An. coluzzii* (p < 0.001, Table 1), but explained < 6% of variation in swarm end times. For *An. gambiae*, temperature and light intensity were highly correlated with the end of swarming (P < 0.0001, Table 1), but each of these parameters could explain only <13% variation, and humidity could explain only 2.4% of variation (p < 0.05, Table 1).

**Table 1:** Regression analysis of swarm start and stop times against environmental parameters based on Pearson’s Correlation Coefficient test (two-tailed distribution)

	***An. coluzzi***		***An. gambiae s.s.***		
	**r**	**p**	**r**	**p**	**df = n-2**
**Start swarm**					
**Temperature**	0.0640	0.3233	0.1694	0.0085	238^&^
**Humidity**	0.4452	< 0.0001	0.2837	< 0.0001	238^&^
**Light intensity**	0.1345	0.0373	0.0412	0.5250	238^&^
**Time of sunset**	0.9606	< 0.0001	0.9481	< 0.0001	498*
**End swarm**					
**Temperature**	0.0735	0.2568	0.2731	< 0.0001	238^&^
**Humidity**	0.2445	<0.001	0.1543	0.0168	238^&^
**Light intensity**	0.1187	0.0663	0.3592	< 0.0001	238^&^
**Time of sunset**	0.9469	< 0.0001	0.9621	< 0.0001	398^#^

The correlation (r) between each environmental variate in turn against swarm start and end times for each species; 'p’ = probability of no correlation, sample size for each species n = 240 records each of temperature, humidity and light intensity at the start and at the end of swarms (^&^20/month/species for 12 months, 2007), n = 500 records of sunset times for start of swarms (*20/month/species for 25 months over 4 years), and n = 400 records of sunset times for the end of swarms (^#^20/month/species for 20 months over 3 years). NB: swarm end times were not recorded in 2009.

**Figure 4:**
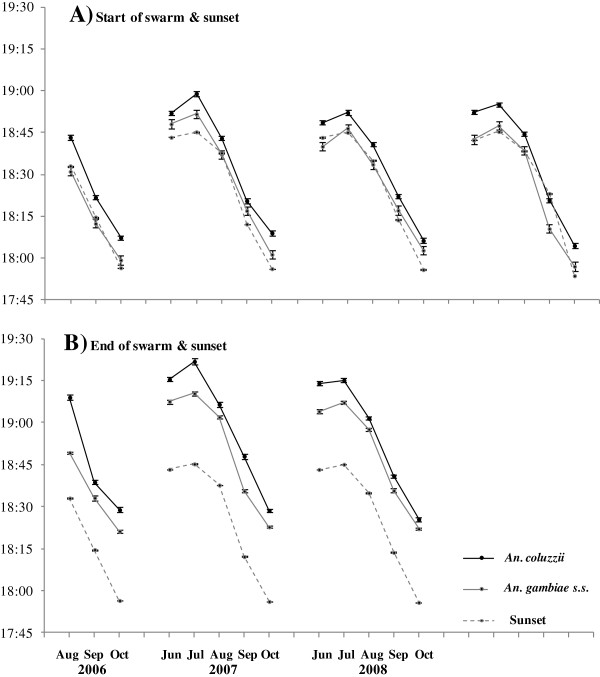
**Relative timing of sunset and the start and end of swarming for *Anopheles coluzzii *and *An. gambiae s.s. *males across four rainy seasons. A)** Start of swarming and sunset, **B)** End of swarming and sunset. Mean ± s.e. times for *An. gambiae s.s.* normalised to sunset times in VK7 to take into account the effect of the difference in latitude and longitude between the two monitoring sites (VK7 and Soumousso) on the 'clock times’ of sunset and the start and end of swarming (see Data analysis test). For each data point, n = 20 (for each species, the same five swarm sites monitored on four consecutive days each month). Note: the end of swarming was not recorded for 2009.

**Figure 5:**
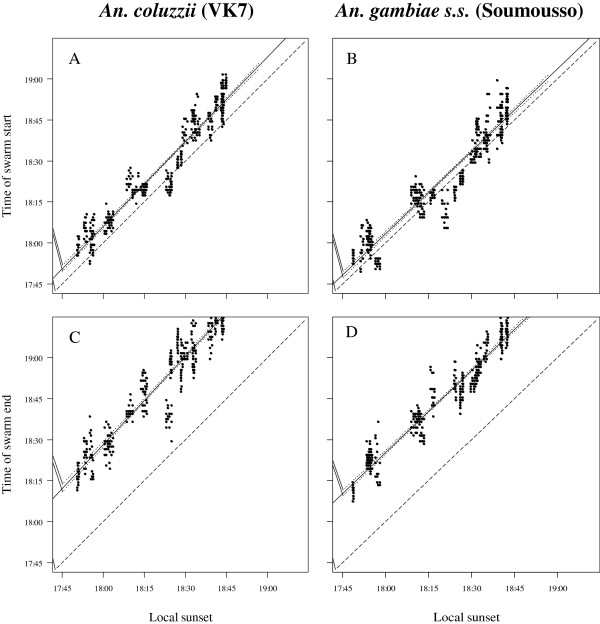
**Regression of time of swarming on time of sunset for *Anopheles coluzzii *in VK7 and *An. gambiae s.s *. in Soumousso. A)***An. coluzzii* start of swarming, **B)***An. gambiae s.s.* start of swarming, **C)***An. coluzzii* end of swarming and **D)***An. gambiae s.s.* end of swarming; solid line = regression, fine dashed line = 95% confidence interval of the regression and coarse dashed line = 1:1 slope for time of sunset (i.e., the distance between this line and the regression line indicates the time after sunset at which swarming started or ended) and n = 240 for each of the four plots.

For both species, however, sunset was by far the parameter most strongly correlated with the timing of the start and end of swarming in 2007. Swarming began close to sunset in most months, except in May, June and July when it occurred noticeably later than sunset during the time of year when day length was rapidly increasing and the time of sunset was delayed to its latest time for the year (Figure 2C). Likewise, changes in the timing of the end of swarming closely followed changes in sunset times (Figure 3C). Similar correlations between the time of sunset and swarming were also evident for swarms monitored during the rainy seasons of 2006, 2008 and 2009 (Figure 4). Regression analyses of all data collected from 2006–2009 for swarming start times (Figure 5 A&B) and end times (Figure 5 C&D) on the concurrent times of local sunset found a highly significant correlation between swarm times and sunset for both species (p < 0.0001, Table 1). The timing of sunset explained > 90% of the variation in the start and end of *An. coluzzii* and *An. gambiae* swarming.

The patterns of variation in data points above and below the regression lines in Figure 5 reflect monthly variations in swarm times, as is also evident in Figures 2C and 3C. The effect of month has not been analysed in depth since we have only one month’s data for November, December and January-May. Nonetheless, the 95% confidence interval lines (fine dashed lines, Figure 5) show, overall, how tightly distributed the data are along the regression lines in all four cases.

### Differences in swarming times between species

Figure 5 shows the relationship between *An. coluzzii* and *An. gambiae* swarm start and end times in relation to their respective local sunset times. For each plot in Figure 5, the time interval between the regression line for swarm time on sunset time (solid line) and the 1:1 line for sunset time on itself (course dashed line) indicates the mean difference in time between the start or end of swarming and sunset. The distances between these two lines in Figure 5A and B suggest that *An. coluzzii* started swarming later with respect to sunset than *An. gambiae*, and a comparison of Figure 5C and D show the same for swarm end times. The mean monthly differences in the offset between sunset and swarm start or end times for the two species were compared by Welch’s *t*-test (unequal variances). *Anopheles coluzzii* started swarming at a mean of ± s.e. 6:76 ± 0:26 min:sec after sunset, significantly later than *An. gambiae*, which started swarming at a mean of 3:41 ± 0:26 min:sec after sunset (p < 0.0001, Table 2). *Anopheles coluzzii* stopped swarming at a mean of 29:85 ± 0:29 min:sec after sunset, significantly later than *An. gambiae*, which stopped swarming at a mean of 25:34 ± 0:29 min:sec after sunset (p < 0.0001, Table 2).

**Table 2:** Comparisons of the mean times (minutes) of swarming activity in relation to sunset

	***An. coluzzi***		***An. gambiae s.s.***				
	**mean**	**se**	**mean**	**se**	**t-test**	**df**	**p**
**Offset between swarm start and sunset**	6.76	0.26	3.41	0.26	9.2515	979.98	<0.0001
**Offset between swarm end and sunset**	29.85	0.29	25.34	0.29	11.0077	752.92	<0.0001
**Swarm duration**	21.39	0.33	23.37	0.33	4.1444	775.62	<0.0001

N = 500 for swarm start times, and 400 for swarm end times and swarm duration (see details in Table 1 legend). Note swarm end times were not recorded for 2009.

**Figure 6:**
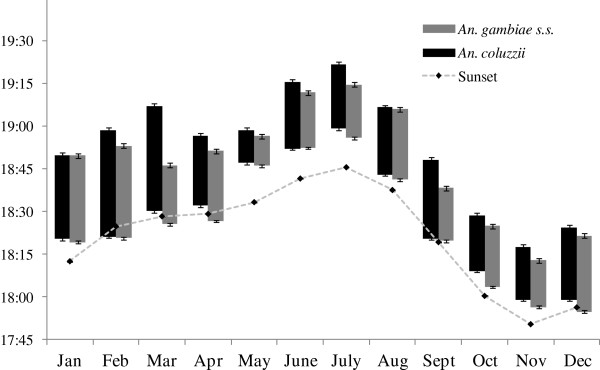
**Duration of male swarms for *Anopheles coluzzii *in VK7 and *An. gambiae s.s.*in Soumousso.** Mean ± s.e. times of the start and end of swarming (i.e., tops and bottoms of bars, respectively) in relation to the time of sunset each month in 2007 (n = 20 swarms/species/month). The 'duration’ time of a swarm is the vertical length of each bar, based on the time scale of the y axis. Sunset and swarm times for Soumousso normalised to VK7 'clock’ times (see Figure 4 legend and Data analysis text). For sunset, s.e. = height of plotted points.

On this basis, and with some reservations, *An. gambiae* can be said to have started swarming a mean of 3:35 ± 0:68 min:sec before *An. coluzzii*, and stopped swarming a mean of 4:51 ± 1:21 min:sec before *An. coluzzii*. These times may seem relatively insignificant; however, given that the mean duration of swarming for *An. coluzzii* males each evening was 21:39 ± 0:33 min:sec (Table 2), they swarmed without *An. gambiae* males present for a mean of 19.5 ± 4.55% of the total duration of their swarming time. The mean duration of swarming for *An. gambiae* males was significantly longer by ~ 2 min (23:37 ± 0:33 min:sec, p < 0.0001, Table 2), and they swarmed without *An. coluzzii* males present for a mean of 15.3 ± 3.08% of the total duration of their swarming time. These data must be interpreted with caution since the accuracy of these estimated times is diminished by each mathematical manipulation. Nonetheless, the general relationship between the timing of swarming in *An. coluzzii* and *An. gambiae* males, as shown in Figure 6 for 2007 (indexed to sunset times in VK7), shows that there is a significant difference between the start and/or end of swarms of the two species for most months of the year. The mean monthly start and stop times for each species are shown, with the duration of swarming filled with solid black for *An. coluzzii* and grey for *An. gambiae*. The duration of swarming was most consistent during the months when populations densities are generally highest (June to October), which are the months for which the most data were collected. It was considered that there were not enough data across all months to analyse further variation in swarm duration between months.

### Onset of spontaneous activity at dusk in *An. coluzzii* and *An. gambiae* males

**Figure 7:**
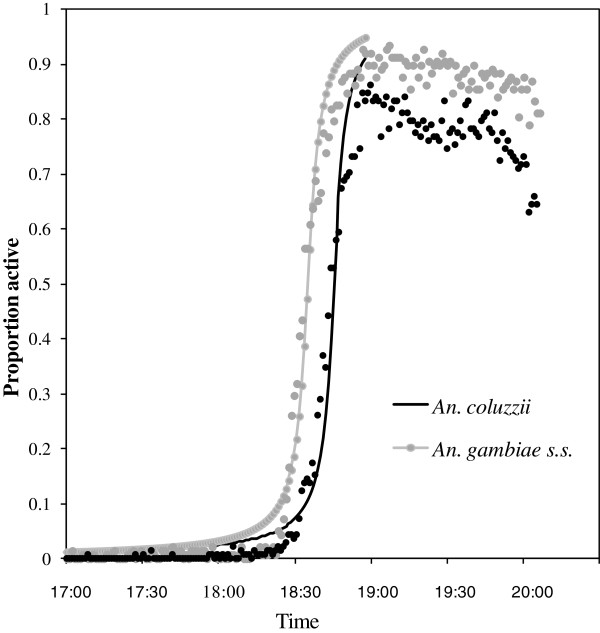
**Comparison of the timing of onset of general dusk activity for male *Anopheles coluzzii *and *An. gambiae s.s.*in an actograph, September–October 2008.** Cauchit curve fitted to data points for each species. Each data point indicates the proportion of males active in that minute, with n = six males of each species/night x 21 nights, i.e., 126 males of each species each minute, over 3 hours, i.e., 180 min).

In total, 126 records were collected over 21 evenings for each species. *Anopheles gambiae* males can be seen to have become active earlier than *An. coluzzii* males in the actograph experiment (Figure 7). The criteria used to define the onset of activity for each group of mosquitoes was the first minute within which more than half the individuals of each species (i.e., at least 4 males) were active, which occurred at 18:32:30 ±00:22 hr:min:ss (mean ± se) for *An. gambiae* males and 09:35 min:sec later at 18:42:05 ± 21 for *An. coluzzii* males, a highly significant difference (ANOVA; F = 293.5, df = 228, p < 0.0001, quasibinomial, cauchit distribution in R). Ambient light intensity had no significant effect on the onset of spontaneous activity (ANOVA; p = 0.8104).

These results are consistent with the field observations that male *An. gambiae* start swarming earlier than *An. coluzzii* males (Results above, Figure 4A). The criteria used to define the start of activity in the two assays are too different, however, to allow more detailed comparisons between the swarm start times and actograph activity times; the start of swarming is defined as the time that the first mosquito started swarming at a particular site, and the start of flight activity in the actograph is defined as the first minute in which more than half the individual mosquitoes of a given species were active. It is worth noting, however, that the actograph assessment compared the activity levels of both species in the same apparatus at the same time, and, therefore, direct comparisons between the species are more reliable.

## Discussion

The occurrence of dusk swarms in *An. coluzzii* and *An. gambiae* appears to be controlled predominantly by time of day. Most reports on the timing of *An. gambiae* swarms in the literature state that swarms form within ~ 10 min of 18:00 [36-39,51]. The field data presented here shows that the time of sunset is the environmental factor that is most strongly correlated with the start and end of swarming activity. Taken together with laboratory-based evidence that male and virgin female *An. gambiae* have a circadian peak in activity during the first hour after lights off [41], the findings presented here suggest that the timing of swarming activity in *An. gambiae s.l.* species is mainly regulated by a biological clock that enables them to adjust to cyclic changes in day length (photoperiod) and the timing of sunset throughout the year. This circadian rhythm of activity enables the synchronisation of male and female sexual activation even as day length varies throughout the year. This observation is not new, as a range of physiological processes and behavioral activities have been shown to be under circadian control in organisms from bacteria to mammals.

Humidity was also significantly correlated with the start and end of swarming. It is worth noting, however, that changes in mean humidity closely followed changes in the timing of sunset; both increased from April-July and decreased from July-December, and, therefore, it is not possible, based on this data set alone, to determine the extent to which changes in humidity have a direct affect on swarming, or are simply correlated with other seasonal changes, such as sunset. Temperature and light intensity at the time of swarming were also significantly correlated with swarming in some cases (Table 1), but unfortunately, changes in these parameters were not measured during the period at dusk leading up to swarming, so it is not possible to determine the extent to which short-term changes in these parameters at sunset might have had an effect on the timing of swarming, or whether changes in these parameters are simply correlated with the overall changes in meterological conditions at dusk, and, therefore, are an alternative way of assessing the correlation of swarming times with sunset. Likewise, it is beyond the scope of this study to distinguish the subtle effects of changes in each environmental parameter over a scale of months since all of these parameters are correlated with seasonal meteorological changes. It is clear, however, that the timing of swarming was not 'triggered’ or initiated in some way by particular values of temperature, humidity or light intensity, or we would have recorded more constant values of these parameters across the seasons and irrespective of changes in sunset times.

Our results also show that swarming began earlier in *An. gambiae* than in *An. coluzzii*, with a mean difference in the start of swarming of ~ 3.5 min, and ended earlier by a mean of ~ 5 min, over the course of a swarming period that lasted for ~22 min in each species. The difference in swarming times of the two species suggest that, when the two species swarm at the same site, each species would be allochronically (i.e., temporally) isolated from the other for ~ 15- 20% of their respective swarm durations. The accuracy of these estimates is compromised by two sources of error due to the limitations of fieldwork; 1) the observations of each species were made in separate villages, separated by ~24' latitude and ~22’ longitude, which means that clock times are not directly comparable, and 2) the timings of the start and end of swarming are based on the appearance of the very first and very last male to be seen swarming over a marker, and therefore subject to variation between individuals as well as between species. The first limitation is, perhaps the most serious one, in that the times of sunset according to a clock would be earlier in Soumousso, where *An. gambiae* swarms were monitored, than in VK7, where *An. coluzzii* swarms were monitored, due to the former being further east and south than the latter. Hence, the swarm start and end times for the two species are not independent of their respective geographic positions. However, the actograph results are more robust because both species were recorded on the same nights under identical conditions, and the differences in timing of activity are based on the mean behaviour of six mosquitoes per data point. The actograph findings indicate that the difference between the two species in onset of activity is ~ 10 min, which implies an even greater degree of allochronic separation between the species in mixed swarms. Thus, the differential timing of swarming in the two species may have contributed to the reproductive isolation of these two taxa, a hypothesis that was proposed by Charlwood and Jones [51] in relation to speciation within the *An. gambiae* complex more than 30 years ago.

A recent laboratory study by Rund *et al*. [52] on differences in the timing of daily activity in M (*An. coluzzii*) and S (*An. gambiae*) molecular form males from laboratory-reared colonies of strains that originated in Mali found that the peak in male M form occurred 2.9 – 4.1 min earlier than in S form males, whereas the study presented here found that *An. gambiae* were active earlier than *An. coluzzii*. Although at first sight these results appear to be contradictory, it is interesting to note that the order of magnitude of difference in the timing of activity is similar (3 – 5 min) in both cases and that the Rund *et al*. study [52] was conducted with strains of mosquito that had been colonised for many generations. The timing of flight activity may be relatively labile within a range of changes of up to 5 minutes, and that the direction of selection for early or late onset of activity may be fairly arbitrary.

Both studies confirm that the timing of mating is predominantly under circadian control, and based on a range of studies in *Drosophila* spp and other insect species [53-55] differences in the phase of activity may lead to temporal separation, and thereby contribute an allochronic component to speciation.

## Conclusions

We have shown that swarming in *An. coluzzii* and *An. gambiae* lasts ~ 20 min at dusk, and that the start and end of swarming in two species occurs at significantly different times; should they swarm at the same site, they would overlap for only 15-20% of the their respective swarming periods. These results support the hypothesis that the two species swarm at partially different times, and, when considered together with their differences in swarm site preferences [21,37] and seasonal differences in population sizes, the overall effect is a reduced likelihood of hybridisation. The implications of these findings are of relevance to the basic concept of releasing genetically modified mosquitoes or sterile males to interfere with reproduction in *An. gambiae* populations; we have shown that the window for mating in swarms is short, and relatively small differences in the timing of activity can significantly reduce the mating competitiveness of colonies reared for release. In addition, the findings presented here contribute to a greater understanding of the prezygotic barriers to hybridisation between *An. coluzzii* and *An. gambiae*.

The success of a GMO or sterile release programmes will depend largely on whether released males can survive and successfully compete for mates against wild males [56-59]. The use/release of such GMO or sterile males requires a proper understanding of potential interactions with naturally occurring populations. Gathering information on the behavioural and ecological factors that govern *Anopheles* reproductive biology in general and in particular regarding swarming and mating behaviour, will increase the chances of success of GMO and sterile male-based control efforts [57-60].

## Competing interests

The authors declare that they have no competing interests.

## Authors’ contributions

SPS conducted the field work and the actograph experiments, analysed the data and participated in drafting the manuscript. CC and AD participated in designing the study and revising the manuscript. CP participated in actograph data analyse. GG designed the actograph experiment, prepared the apparatus and contributed to data analysis and drafting the paper, RKD participated in designing the study, supervised the field work, analysed the data and wrote the paper. All authors read and approved the final version of the manuscript.
